# Nutcracker Syndrome: A Rare Cause of Hematuria

**DOI:** 10.7759/cureus.75777

**Published:** 2024-12-16

**Authors:** Rafaela Lopes Freitas, Inês Carqueja, Sara Rocha

**Affiliations:** 1 Internal Medicine, Pedro Hispano Hospital, Matosinhos, PRT; 2 Intensive Care, Pedro Hispano Hospital, Matosinhos, PRT

**Keywords:** hematuria, internal medicine, left renal vein compression, nutcracker syndrome, urology

## Abstract

Nutcracker syndrome (NCS) is an uncommon vascular condition caused by the compression of the left renal vein (LRN), which may result in venous hypertension and clinical symptoms, including hematuria, flank pain, and pelvic congestion. This report describes the case of a 30-year-old woman with recurrent painless macroscopic hematuria, ultimately diagnosed with NCS based on clinical and radiological findings. Computed tomography (CT) angiography revealed a reduced aortomesenteric angle and LRN compression without signs of severe venous hypertension or collateral circulation. Conservative management was successfully adopted, with no complications reported over three years of follow-up. This case highlights the importance of distinguishing anatomical findings from clinically significant syndromes and supports non-invasive management in mild presentations.

## Introduction

Nutcracker syndrome (NCS) is a rare clinical condition defined by left renal vein (LRV) entrapment [1]. Although its prevalence remains uncertain, it predominantly affects women and can occur at any age [2]. NCS is classified as anterior (compression between the abdominal aorta and the superior mesenteric artery (SMA)) or posterior (compression of a retro-aortic or circum-aortic renal vein between the aorta and vertebral body) [3].

The physiological aortomesenteric angle ranges from 40° to 90°, and reductions of this angle can lead to LRV hypertension causing a spectrum of clinical manifestations ranging from asymptomatic cases to severe presentations, such as hematuria (the most common), pelvic pain, orthostatic proteinuria, and varicocele in male patients [4]. Ultimately, chronic renal disease and renal vein thrombosis can occur [5,6].

Despite its well-described pathophysiology, NCS remains a diagnostic challenge due to its rarity and the nonspecific nature of its symptoms, often mimicking other urological or nephrological conditions. This report aims to highlight the diagnostic approach, management, and outcomes in a patient diagnosed with NCS, emphasizing the importance of high suspicion for vascular anomalies in patients presenting with persistent or recurrent hematuria of unclear origin to mitigate potential complications and improve patient outcomes.

## Case presentation

A healthy 30-year-old woman presented to the emergency department with recurrent episodes of painless macroscopic hematuria. The patient described three similar episodes of dysuria and macroscopic hematuria, without any other associated symptoms in the preceding months. "She was empirically treated twice for a urinary tract infection.

**Figure 1:**
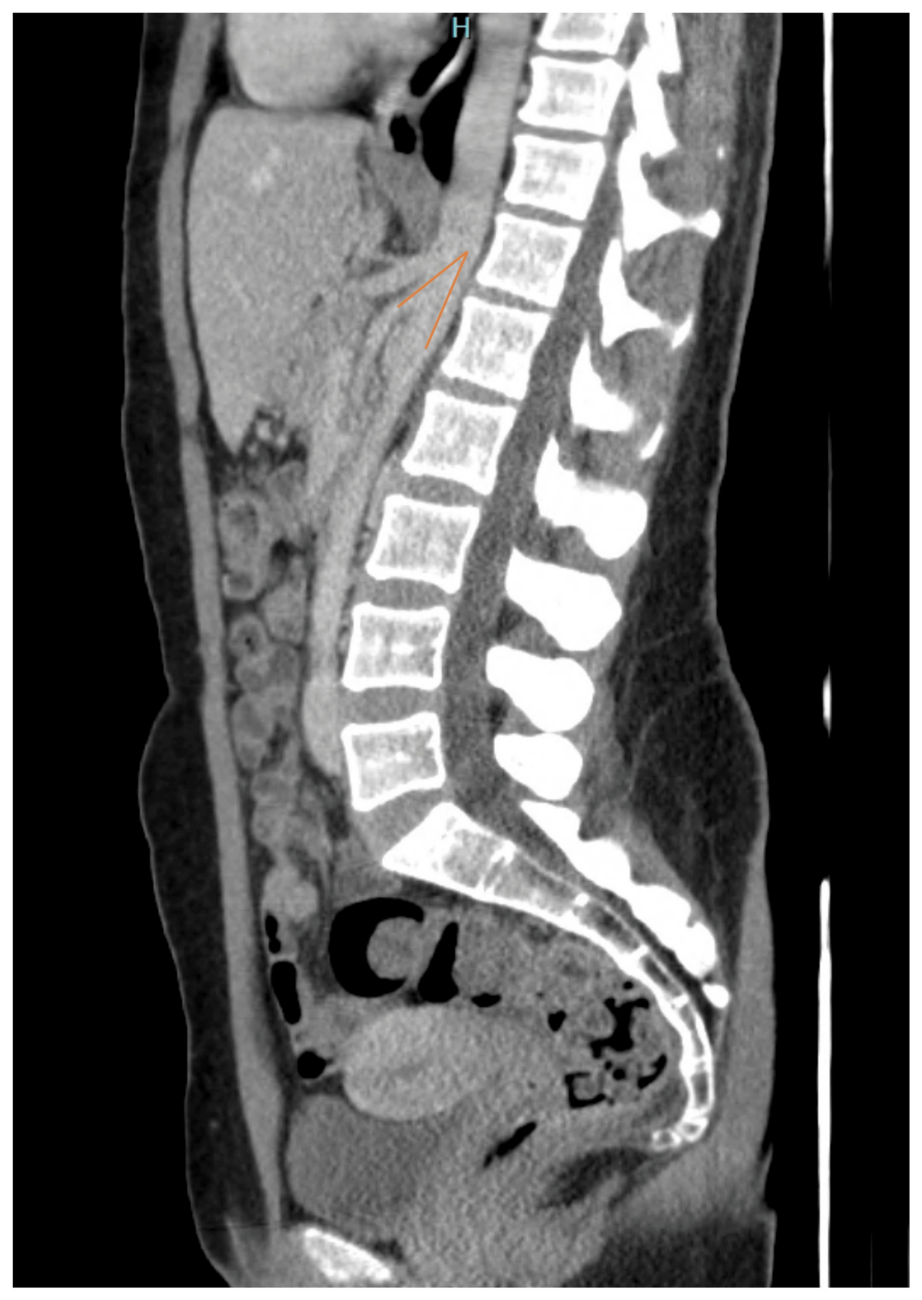
A sagittal view of the CT scan shows the origin of the SMA (left arrow) and the aorta (right arrow), with the compressed LRV in the narrowed space. CT, computed tomography; SMA, superior mesenteric artery; LRV, left renal vein

**Figure 2:**
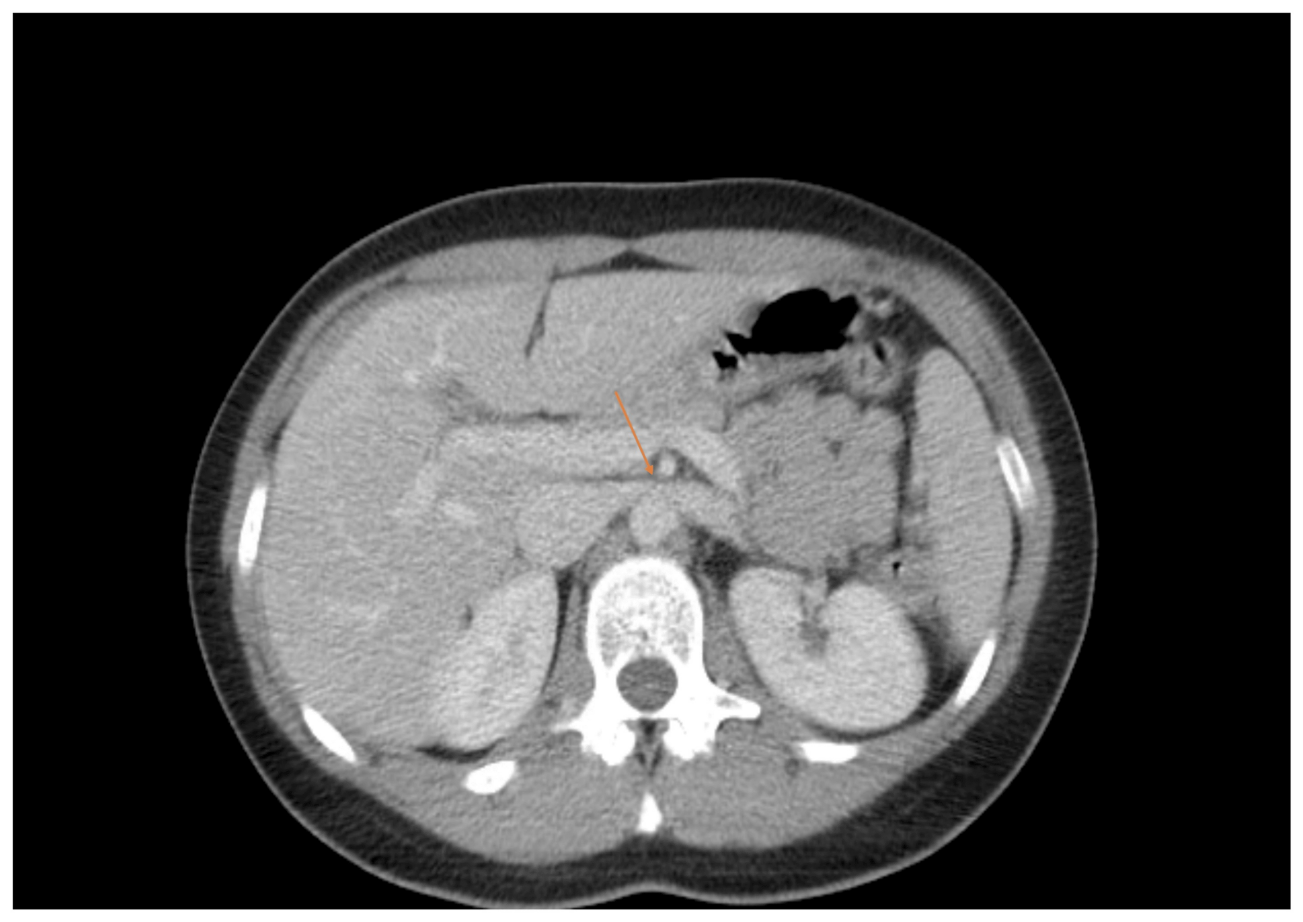
CT showing compressed LRV. CT, computed tomography; LRV, left renal vein

Physical examination was unremarkable. Body mass index (BMI) was 24 kg/m^2^. Laboratory tests revealed a creatinine level of 0.8 mg/dL (0.7-1.3 mg/dL); urinalysis showed mild hematoproteinuria. The urine culture showed no signs of infection. Abdomino-pelvic contrast-enhanced computed tomography (CT) demonstrated a reduced angle between the aorta and SMA of approximately 15° with compression of the LRV (Figure 1 and Figure 2). No collateral vessels or gonadal reflux were observed.

Other potential causes such as urinary tract infections, urolithiasis, nephrolithiasis, neoplasms, endometriosis, and glomerulonephritis were ruled out (Table 1).

**Table 1:** Laboratory results on admission in this case of NCS. The table outlines key laboratory findings, including renal function tests and urine analysis. NCS, nutcracker syndrome

Analysis	Result	Reference range
Hemoglobin	13.2 g/dL	13-18 g/dL
White blood cells	9.87x10^3 ^u/L	4-11x10^3 ^u/L
Platelets	230x10^3 ^u/L	150-400x10^3 ^u/L
Urea	25 mg/dL	18-55 mg/dL
Creatinine	0.8 mg/dL	0.7-1.3 mg/dL
Sedimentation rate	10 mm/h	<20 mm
Urine analysis	150 erythrocytes/field (++), proteins (+), nitrites negative, leukocytes negative	

The patient was diagnosed with NCS, and a conservative approach was adopted with annual imaging (specifically abdominal-pelvic Doppler ultrasound) and renal function assessments. Over a three-year follow-up period, the patient remained asymptomatic, with no complications reported.

## Discussion

NCS is a rare but likely underdiagnosed cause of recurrent hematuria, resulting from LRV hypertension due to extrinsic compression. Prevalence and natural history are still poorly understood.

In this case, the authors present a 30-year-old woman with periodic macroscopic hematuria who was diagnosed with NCS based on clinical and radiological criteria. CT angiography revealed compression of the LRV with a reduced aortomesenteric angle but without signs of severe venous hypertension or significant clinical repercussions.

According to the 2024 Consensus on NCS [1], the diagnosis requires a combination of clinical symptoms, radiological findings, and exclusion of secondary causes of hematuria.

Advances in diagnostic modalities, including Doppler ultrasound, CT, and magnetic resonance imaging (MRI), have significantly enhanced the precision in identifying NCS. In fact, Doppler ultrasound remains a valuable first-line diagnostic tool due to its capability to measure peak velocity ratios in the LRV. In contrast, CT and MRI provide superior spatial resolution, allowing for the detailed assessment of venous compression and collateral circulation, which are crucial in the diagnostic workup of NCS [7].

Despite these advancements, retrograde venography continues to be regarded as the gold standard for confirming elevated venous pressures. However, its use is typically reserved for patients requiring further invasive intervention [2].

Anatomical compression is relatively common in imaging studies, especially in thin individuals, although only a minority of cases present clinically significant repercussions. Furthermore, the benign findings on CT underscore the importance of distinguishing functional compression of the renal vein from a truly pathological clinical condition. As observed in this case, the interpretation of radiological findings must be done cautiously, considering their correlation with the patient's symptoms.

Management depends on clinical presentation, severity of the symptoms, impact on quality of life, and prognosis. Treatment options range from expectant surveillance and endoscopic procedures (stenting or hemostatic agents) to more complex surgical procedures (LRV transposition or renal autotransplantation) [8,9]. Endovascular stenting is discouraged as the primary option due to the high risk of complications like stent migration [1].

Conservative clinical monitoring and periodic re-evaluation are recommended for cases with mild to moderate symptoms, as observed in this patient. The 2024 NCS consensus emphasizes weight gain in underweight patients (BMI<18.5 kg/m²) as the first-line approach [1], although our patient was within the normal weight range. Nevertheless, there is no data on the optimal duration of follow-up.

## Conclusions

This case underscores diagnostic and management complexities associated with NCS, particularly in patients presenting with benign radiological findings and mild symptoms. The conservative approach adopted in this case effectively prioritized non-invasive management, which is increasingly recognized as appropriate for patients with mild clinical presentations and stable disease. Distinguishing between incidental anatomical findings and clinically significant conditions remains critical to avoid unnecessary diagnostic or therapeutic interventions. Standardized guidelines, informed by recent advances in imaging and therapeutic modalities, are imperative.
